# Prematurely aging mitochondrial DNA mutator mice display subchondral osteopenia and chondrocyte hypertrophy without further osteoarthritis features

**DOI:** 10.1038/s41598-020-58385-w

**Published:** 2020-01-28

**Authors:** Jeroen Geurts, Sonia Nasi, Pascal Distel, Magdalena Müller-Gerbl, Tomas A. Prolla, Gregory C. Kujoth, Ulrich A. Walker, Thomas Hügle

**Affiliations:** 1 Department of Rheumatology, Lausanne University Hospital (CHUV), Lausanne, Switzerland; 2 Department of Biomedical Engineering, University Hospital of Basel, Basel, Switzerland; 3 Department of Anatomy, University of Basel, Basel, Switzerland; 4 Departments of Genetics and Medical Genetics, University of Wisconsin, Madison, USA; 5 Department of Rheumatology, University Hospital of Basel, Basel, Switzerland

**Keywords:** DNA damage and repair, DNA damage and repair, Bone, Bone

## Abstract

Mitochondrial mutations and dysfunction have been demonstrated in several age-related disorders including osteoarthritis, yet its relative contribution to pathogenesis remains unknown. Here we evaluated whether premature aging caused by accumulation of mitochondrial DNA mutations in *Polg*^*D*2*75A*^ mice predisposes to the development of knee osteoarthritis. Compared with wild type animals, homozygous *Polg*^*D275A*^ mice displayed a specific bone phenotype characterized by osteopenia of epiphyseal trabecular bone and subchondral cortical plate. Trabecular thickness was significantly associated with osteocyte apoptosis rates and osteoclasts numbers were increased in subchondral bone tissues. While chondrocyte apoptosis rates in articular and growth plate cartilage were similar between groups, homozygous mitochondrial DNA mutator mice displayed elevated numbers of hypertrophic chondrocytes in articular calcified cartilage. Low grade cartilage degeneration, predominantly loss of proteoglycans, was present in all genotypes and the development of osteoarthritis features was not found accelerated in premature aging. Somatically acquired mitochondrial DNA mutations predispose to elevated subchondral bone turnover and hypertrophy in calcified cartilage, yet additional mechanical or metabolic stimuli would seem required for induction and accelerated progression of aging-associated osteoarthritis.

## Introduction

Mitochondrial dysfunction and DNA (mtDNA) mutations in articular chondrocytes has gained increasing interest as a pathophysiological mechanism underpinning development of aging-associated osteoarthritis (OA)^1–4^. Mitochondrial dysfunction can be induced in chondrocytes by both mechanical^2^ and inflammatory stimuli^3^ and in turn promotes the production of reactive oxygen species (ROS) and the induction of apoptosis and cell death. Selective removal of damaged and dysfunctional mitochondria from OA chondrocytes under pathological conditions results in diminished ROS levels and inhibition of apoptosis^3^. Lower chondrocyte apoptosis rates in specific mtDNA haplotypes have been associated with a lower risk of incident knee OA in prospective cohort studies^1,5^. However, the evidence linking mtDNA mutations to OA susceptibility has been demonstrated in *ex vivo* studies only. While the accumulation of mtDNA mutations *in vivo* has been shown to affect musculoskeletal tissues and induce premature aging^6,7^, the joint phenotype has not been evaluated thus far. Mice with defective DNA damage repair did not show accelerated OA development during premature ageing, despite elevated turnover of subchondral bone tissues^8^.

Mice carrying a homozygous homozygous proof-reading deficient version of the mtDNA polymerase gene *Polg* are characterized by a reduced life span, with a maximum survival of 15 months, and progeroid features such as sarcopenia and kyphosis that become apparent from the age of 9 months^6,7^. Increased apoptosis rates, but not ROS production, between the age of 3 and 6 months in tissues with rapid cellular turnover has been identified as the pivotal mechanism underpinning the premature aging phenotype. Postmitotic tissues, including cartilage and bone, displayed increased tissue apoptosis at later time points and tibial bone mineral density was found decreased from the age of 10 months^7^.

In the present study, we assessed degeneration of subchondral bone and articular cartilage tissues in knee joints of prematurely aging homozygous *Polg*^*D275A*^ mtDNA mutator mice in comparison with heterozygous mutants and wild type littermates.

## Methods

This is a descriptive study using a convenience sample, therefore no statistical methods were used to predetermine sample size. The experiments were not randomized and investigators were not blinded to allocation during experiments and outcome assessment.

### Mutator mice

The generation and phenotypic characterization of *Polg*^*D275A*^ mtDNA mutator mice used for this study has been described in detail elsewhere^7^. Homozygous mutants develop progeroid features at 9 months of age and the maximum life span is reduced to 15 months. The convenience sample used in this descriptive study comprised three sets of littermates: Male wild type and homozygous mutants aged 11.3 and 12.2 months. Male wild type, homozygous and heterozygous (*n* = 2) mutants aged 14.5 months. Two female and one male wild type and heterozygous aged between 14.5 and 15.7 months. In total, three homozygous mutants, five heterozygous and six wild type mice were analysed. The study protocol was approved by the Animal Care and Use Committee of the University of Wisconsin-Madison, USA. Experiments were performed in accordance with institutional guidelines and regulations.

### Micro computed tomography scanning and analysis

Knee joints were dissected and fixed in formalin for 24 hours. Subsequently, samples were transferred to 70% ethanol and X-ray scanning was performed using a benchtop micro computed tomography (μCT) scanner (Skyscan 1275, Bruker, Kontich, Belgium) using the following parameters: 50 kV, 200 μA, 50 ms exposure time, 8 μm resolution. Quantitative analysis of tibial and femoral subchondral cortical bone and epiphyseal trabecular bone parameters was performed using CTAnalyzer Version 1.10 (Bruker). Subchondral cortical plate and epiphyseal trabecular bone were segmented manually. Bone volume fraction (BV/TV), bone mineral density (BMD), trabecular thickness (Tb.Th) and trabecular number (Tb.N) were determined for epiphyseal bone. BMD and subchondral cortical plate thickness (SCP.Th) were calculated for cortical volumes of interest. Data are expressed as percentages of corresponding wild type controls. Tibial diameter was measured on a transaxial image at a fixed 1 mm reference point below the tibial growth plate.

### Histology and histomorphometry

Samples were decalcified in EDTA for 14 days and embedded in paraffin. Cartilage damage was assessed by two independent on 6 μm frontal joint sections stained with Safranin-*O*/Fast Green using the OARSI histologic scoring system^9^. Osteoarthritis severity was assessed by determining cumulative OARSI scores over four joint compartments (medial & lateral tibia and medial & lateral femur, maximum score 24).

Osteoclast activity was assessed using tartrate-resistant acid phosphatase (TRAP) staining. Tissue sections were pre-incubated in TRAP buffer (0.1 M acetate buffer pH 5.2, 50 mM sodium tartrate) at 37 °C for 20 minutes. Enzyme reactivity was visualized by incubating sections in TRAP buffer containing 0.1 mg/ml naphthol AS-MX and 0.3 mg/ml fast red violet LB salt at 37 °C for 1 hour. TRAP-stained sections were rinsed in PBS, counterstained with methylene blue and mounted in Vectamount AQ (Vector Laboratories, USA). Bone area fractions and osteoclast numbers (Oc.N) were quantified per mm of bone perimeter using histomorphometry^10^. Briefly, photomicrographs of femorotibial joints were used to generate black-and-white image masks, representing bone and non-bone tissues between epiphyses and articular cartilage. Bone area fractions and perimeter were quantified using ImageJ (Version 1.49q, National Institute of Health, Bethesda, USA). The abundance of hypertrophic chondrocytes in femorotibial cartilage were scored semi-quantitatively in Safranin-*O*-stained tissue sections using the following grades: 0 = none, 1 = <10 hypertrophic chondrocytes per compartment, 2 = 10–20 hypertrophic chondrocytes per compartment, 3 = >20 hypertrophic chondrocytes per compartment.

### Immunohistochemistry analyses

Apoptotic articular and growth plate chondrocytes and subchondral osteocytes were determined in histological tissue sections using an Apoptag kit according to the manufacturer’s instructions (ApopTag Plus Peroxidase *In Situ* Apoptosis Kit, Merck, Darmstadt, Germany). Total and Apoptag-labeled cells were counted by two independent observers. For osteocalcin staining, sections were deparaffinized and endogenous peroxidase activity was blocked in 1.5% H_2_O_2_ for 10 minutes. Primary antibody staining (rabbit anti-osteocalcin, 1:500, (ab93876), Abcam, Cambridge, UK) was performed overnight at 4 °C. Subsequently, sections were incubated in biotinylated goat-anti-rabbit (1:200, Dako, Glostrup, Denmark) for 35 minutes and labeled with a horseradish peroxidase-conjugated biotin-streptavidin kit (Vectastain ABC, Vector Laboratories, Peterborough, UK). Immunoreactivities were visualized using 3,3′-diaminobenzidine as a substrate.

### Statistical analysis

Data for which the normality assumption is tenable are represented as mean ± standard deviation, otherwise dotplot with medians were used. Statistical differences between groups were assessed using one-way ANOVA or Kruskal-Wallis test in GraphPad Prism 6.01 (GraphPad Software, Inc, La Jolla, USA). Spearman’s rank correlation and linear regression were used to evaluate overall association between measurements. P values less than 0.05 were regarded as statistically significant.

## Results

### Homozygous mtDNA mutator mice display osteopenia in femorotibial joints

**Figure 1:**
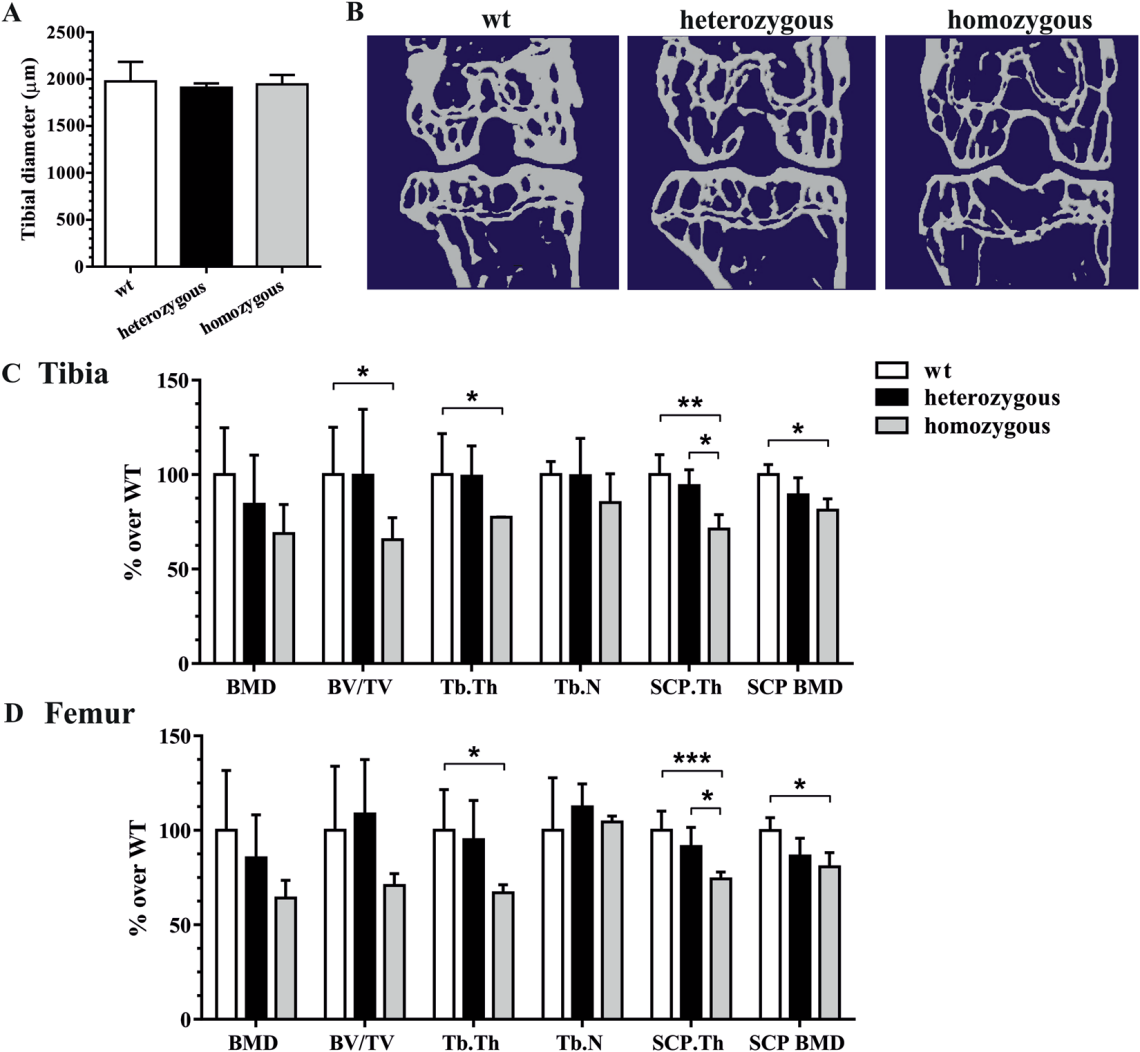
Measurement of tibial diameter at the transaxial reference plane 1 mm below the growth plate (**A**). Representative two-dimensional μCT images of the subchondral cortical plate and epiphyseal trabecular bone of the tibiofemoral joint in wild type, heterozygous and homozygous mutator mice (**B**). Quantitative assessment of trabecular and cortical bone parameters in tibial (**C**) and femoral compartments (**D**). *P < 0.05 and **P < 0.01 by One-way ANOVA.

**Figure 2:**
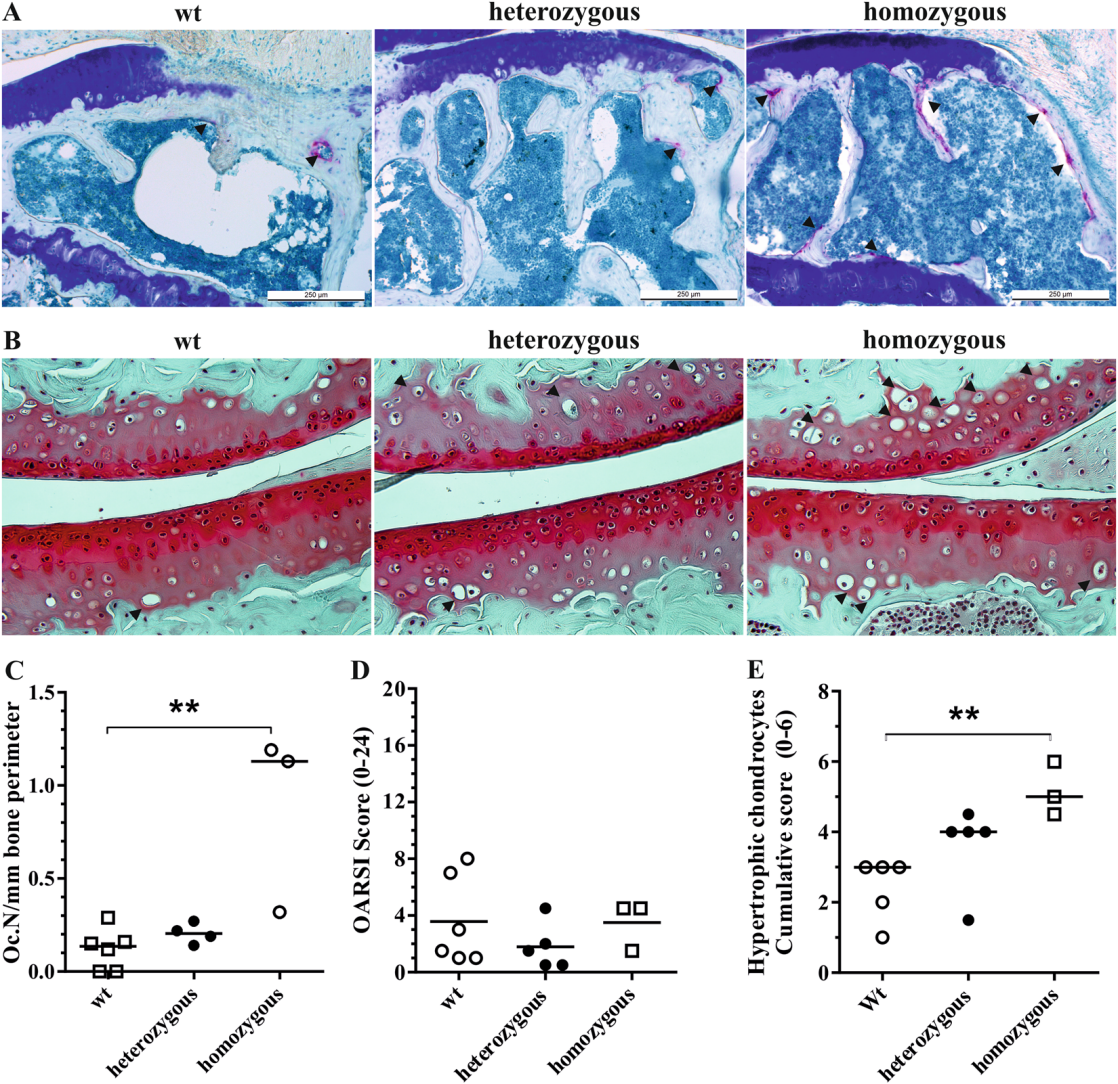
Representative TRAP-stained tissue sections of the epiphyseal bones of the tibial compartment in wild type, heterozygous and homozygous mutator mice (**A**). Increased TRAP-staining (arrowheads) was present at the subchondral cortical plate and trabecular bone of homozygous mutants. Representative images of Safranin-*O*-stained tissue sections of the tibiofemoral joints. Hypertrophic chondrocytes were observed in calcified cartilage (arrowheads) (**B**). Histomorphometric assessment of osteoclast numbers per mm bone perimeter confirmed a significant increase of osteoclasts in homozygous mutator mice compared to wild type littermates (**C**). Cumulative OARSI scores of tibiofemoral joint compartments confirmed a lack of severe OA in all groups (**D**). Numbers of hypertrophic chondrocytes were elevated in tibiofemoral cartilage **(E)** **P < 0.01.

Since homozygous mutant mice displayed loss of body weight and a ten percent reduction of muscle mass from the age of 10 months^7^, we first verified normal skeletal development without changes in tibial size. Regression analysis revealed an overall age-dependent reduction in tibial diameter (r^2^ = 0.67, *p* = 0.007) in all genotypes. The average tibial diameter was similar between wild type mice and heterozygous and homozygous mutants (Fig. 1A). Compared to wild type mice, quantitative μCT analysis revealed a strong reduction of bone volume in tibial and femoral epiphyseal trabecular bone in homozygous, but not heterozygous mtDNA mutator mice (Fig. 1B). In the tibial compartment, BV/TV and Tb.Th were both significantly lower in mtDNA mutator mice compared with wild type mice. In homozygous mutants, the subchondral cortical plate was approximately 30% thinner and its BMD significantly lower if compared to those of wild type mice (Fig. 1C). Reduced SCP.Th and BMD was also observed in the femoral compartment of homozygous *Polg*^*D275A*^ mice. Moreover, femoral epiphyseal bone displayed reduced Tb.Th compared to wild type (Fig. 1D). Osteophyte formation was not observed in any genotype. TRAP staining revealed a significant increase in osteoclast numbers in epiphyseal trabecular bone homozygous mtDNA mutator mice (Fig. 2A,C). Osteocalcin immunohistochemistry showed a diffuse staining pattern in epiphyseal marrow tissue, but no appreciable differences between genotypes (data not shown).

### Elevated chondrocyte hypertrophy, but not cartilage degradation in mtDNA mutator mice

Histological grading of osteoarthritis severity revealed only mild cartilage degeneration, predominantly loss of proteoglycan staining, in tibiofemoral joint compartments of all genotypes (Fig. 2B). Variation in cumulative OARSI scores was not associated with age and median scores did not differ between genotypes (Fig. 2D). In contrast, enumeration of hypertrophic chondrocytes in the calcified cartilage layer showed an increase in homozygous mtDNA mutator mice compared with wild type littermates (Fig. 2B,E). Variation in overall chondrocyte hypertrophy was not correlated with age (*r*^*2*^ = 0.03), suggesting a genotype-specific increase. Articular chondrocytes in calcified cartilage expressed osteocalcin as a posthypertrophic marker^11^, but the percentages did not differ significantly between wild type (10.1 [7.0–25.7] %), heterozygous mutants (11.0 [6.8–17.6]%) and homozygous mutants (19.5 [7.0–24.6]%). The percentage of osteocalcin-positive chondrocytes was not associated with hypertrophic chondrocytes.

### Osteocyte apoptosis correlates with trabecular thickness

**Figure 3:**
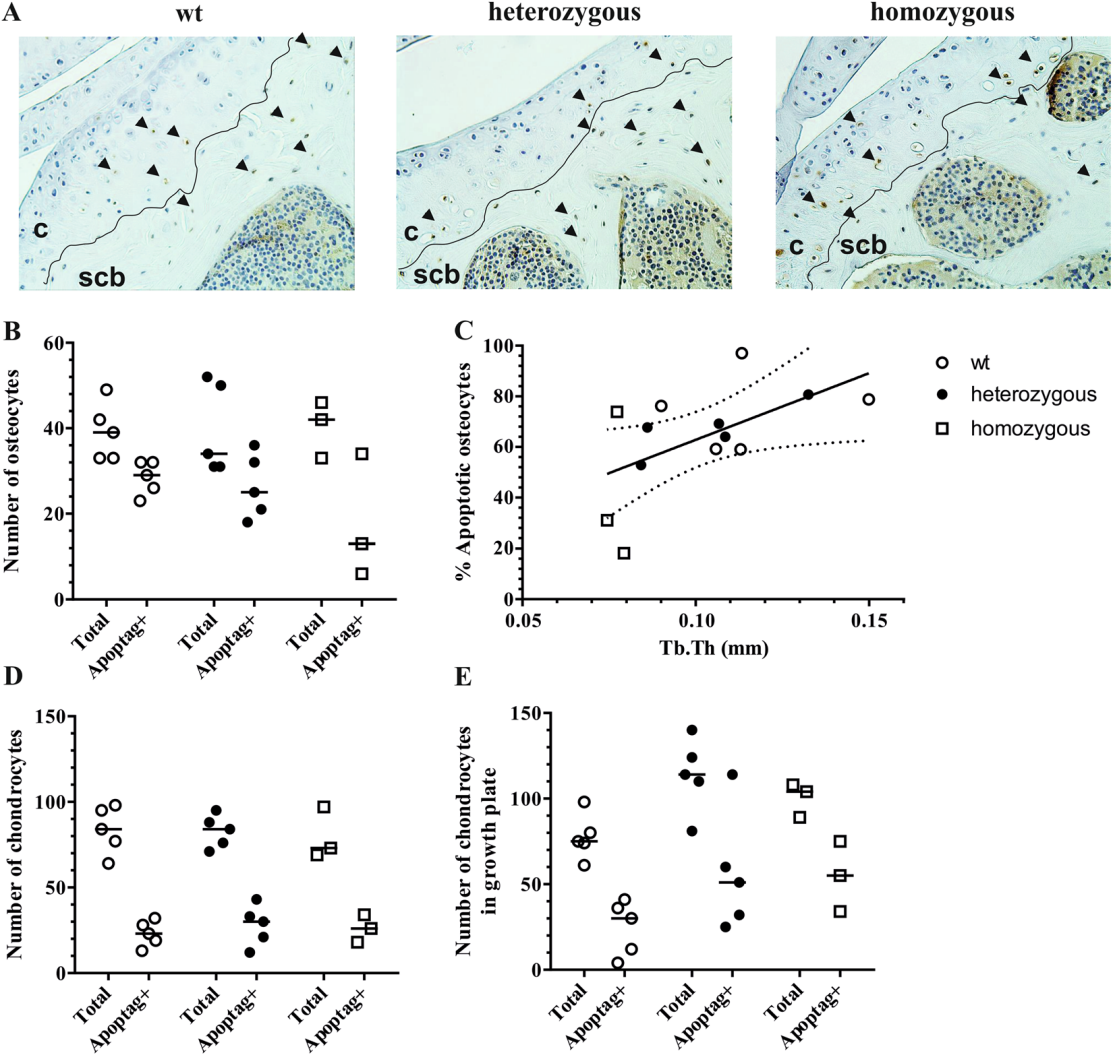
Representative tissue sections stained for apoptotic cells (arrowheads) using an indirect TUNEL method (**A**). Apoptotic (Apoptag+) and total amount of cells were counted in subchondral bone (**B)** articular cartilage **(D)** and growth plate cartilage (**E**). Correlation between femorotibial trabecular thickness measurements and percentage of apoptotic osteocytes (**C**). The regression line with 95% confidence intervals are displayed. **c:** cartilage, **scb**: subchondral cortical bone.

Tissue dysfunction in mtDNA mutator mice has been shown to occur in the absence of ROS production and oxidative stress, and is regulated through an increase in mitochondria-mediated apoptosis^7^. To assess whether apoptosis levels were linked to subchondral osteopenia and chondrocyte hypertrophy, we enumerated apoptotic chondrocytes in articular and growth plate cartilage and osteocytes in epiphyseal trabecular bone using an indirect TUNEL method (Fig. 3A). Osteocyte apoptosis rate were not different between genotypes, yet high variation was found in homozygous mutants (Fig. 3B). However, overall osteocytes apoptosis rates were significantly correlated with trabecular thickness measurements (Spearman *r* = 0.63, *p* = 0.03)(Fig. 3C). Apoptosis rates in articular (Fig. 3D) and growth plate chondrocytes (Fig. 3E) were similar between genotypes (~30%). These data suggest that changes in apoptosis rates in mtDNA mutator mice occurs predominantly in bone, but not cartilage tissues.

## Discussion

This descriptive analysis using a convenience sample of mtDNA mutator mice, characterized by a premature aging phenotype, suggest that accumulation of mtDNA mutations in joint tissues *in vivo* may predispose to osteopenia of subchondral bone and chondrocyte hypertrophy, but not accelerated development of osteoarthritis.

While both mitochondrial dysfunction and accumulation of mtDNA mutations have been associated with increased cellular apoptosis in culture-expanded chondrocytes^1–3^, the impact of both features might be different *in vivo*. Genetically-engineered mice with chondrocyte specific inactivation of the respiratory chain developed postnatal growth retardation and severe growth plate degeneration at 12 months of age, which associated with increased chondrocyte death at the cartilage-bone junction^12^. Mitochondrial respiration was found dispensable for survival of growth plate chondrocytes in fetal development and apoptosis-independent regulation of hypoxia levels appear crucial in early developmental stages^13^. In contrast, homozygous mtDNA mutator mice did not show growth retardation (Fig. 1A) or degeneration of the growth plate (Fig. 3E), which may correspond to the mitochondria-mediated apoptotic pathways that are independent from increased ROS production or oxidative stress in *Polg*^*D275A*^ mice^7^. Mitochondrial dysfunction and mtDNA mutations might therefore regulate chondrocyte survival though distinct mechanisms. It would therefore be interesting to investigate whether chondrocyte-specific inactivation of the respiratory chain led to degenerative changes of articular cartilage.

Increased subchondral bone remodelling favouring osteopenia might be a common feature in premature aging phenotypes caused by accumulation of mtDNA^7^ or DNA damage^8^. The development of typical OA features including structural cartilage degradation and osteophyte formation has not been observed in DNA repair-deficient mice and since the mice analysed here were reaching their maximum life span, severe osteoarthritis is also unlikely to occur in mtDNA mutator mice. The fact that cartilage is a non-vascularized postmitotic tissue with low metabolic activity might render chondrocytes less sensitive to age-related accumulation of mtDNA mutations. To evaluate the clinical relevance of mtDNA haplotypes^1,5^, comparison of experimental osteoarthritis in conplastic mice strains may provide additional insights into functional consequences in cartilage tissues^14^.

The sequential events in subchondral bone and cartilage tissues leading to osteoarthritis remain incompletely understood. In the absence of an additional biomechanical or metabolic stimulus, subchondral osteopenia alone appeared insufficient to promote the development of OA. In contrast, osteoporotic OA is a disease phenotype observed in up to 30% of patients and is characterized by a lack of subchondral bone formation and osteosclerosis^15,16^. A transient increase of osteoclast activity in epiphyseal bone and subsequent thinning of the subchondral cortical plate, as also seen in this study, has been identified as crucial pathomechanism in development of early OA induced by transection of the anterior cruciate ligament in mice and dogs^15,16^. In these surgical models, this initial phase is followed by spatio-temporal uncoupling of bone resorption and subsequent new bone formation in the subchondral bone compartment. For the latter phase, increased transforming growth factor-beta and WNT signalling pathways have been implicated. Molecular pathways, including those involved in chondrocyte hypertrophy, were not investigated in the present study, which comprises one of the limitations. Second, this descriptive study was performed on a convenience sample, which means generalized assumptions about the role of mtDNA mutations in OA cannot be made. Last, male and female mice were not analysed separately. While gender-specific differences in bone structure and physiology have been well established, bone loss in male only mtDNA mutator mice was more pronounced even when comparing with mixed female and male groups.

Taken together, premature aging mtDNA mutator mice display subchondral osteopenia and increased articular chondrocyte hypertrophy in knee joints without additional OA features.
